# Individualized Upfront Treatment Selection for Aneurysmal Subarachnoid Hemorrhage and Functional Outcomes: A Single-Center Retrospective Before-and-After Cohort Study

**DOI:** 10.3390/neurolint18050093

**Published:** 2026-05-15

**Authors:** Atsushi Nakayashiki, Kunihiko Umezawa, Yasuo Nishijima, Ryutaro Suzuki, Michiko Yokosawa, Hidenori Endo

**Affiliations:** 1 Department of Neurosurgery, Iwate Prefectural Central Hospital, Morioka 020-0066, Japan; 2 Department of Neurosurgery, Tohoku University Graduate School of Medicine, Sendai 980-8574, Japan

**Keywords:** aneurysmal subarachnoid hemorrhage, endovascular treatment, microsurgery, individualized modality selection, functional outcome

## Abstract

**Background/Objectives:** The optimal upfront modality selection for real-world aneurysmal subarachnoid hemorrhage (aSAH) remains uncertain. We evaluated outcomes after an institutional change from an endovascular treatment (EVT)-first default to a modality-neutral individualized pathway. **Methods:** This single-center retrospective before-and-after cohort study included consecutive patients with aSAH who underwent aneurysm securing during two fixed time periods (pre-change: 1 May 2023 to 31 July 2024; post-change: 1 August 2024 to 31 October 2025). The primary outcome was a favorable 90-day modified Rankin Scale (mRS) score of 0–2. The primary analysis used Firth penalized logistic regression adjusted for age, pre-morbid mRS ≥ 2, and World Federation of Neurosurgical Societies grade IV–V. Conventional logistic regression and ordinal mRS shift analysis were performed as sensitivity analyses. **Results:** A total of 104 patients were included (pre-change, *n* = 48; post-change, *n* = 56). EVT decreased from 79.2% to 37.5%, and microsurgery increased from 20.8% to 62.5% (*p* < 0.001). Favorable outcomes occurred in 25/48 patients (52.1%) in the pre-change period and 36/56 patients (64.3%) in the post-change period (*p* = 0.235). In adjusted analyses, the post-change period was associated with favorable outcome (aOR 3.82; 95% CI, 1.31–12.79; *p* = 0.009), consistent with the sensitivity analysis (aOR, 4.41; 95% CI, 1.43–15.95; *p* = 0.009). Shift analysis also favored the post-change period (adjusted common OR, 2.36; 95% CI, 1.15–4.91; *p* = 0.021). Secondary outcomes and procedure-related complications were similar between the two periods. **Conclusions:** A shift from an EVT-first default to a modality-neutral individualized pathway was associated with more favorable adjusted 90-day functional outcomes. Multicenter confirmation is warranted.

## 1. Introduction

Endovascular treatment (EVT) has become increasingly common for aneurysmal subarachnoid hemorrhage (aSAH), supported by pivotal randomized evidence demonstrating favorable outcomes in selected patients [1,2]. In many healthcare systems, this trend has accelerated with advances in microcatheters, coils, adjunctive techniques, and periprocedural imaging, as well as the less invasive nature of EVT and its potential for shorter procedural times [3,4]. In contemporary practice, EVT now accounts for a substantial proportion of aneurysm-securing procedures worldwide; notably, in the international phase 3 REACT trial, 70.0% of patients underwent endovascular coiling, compared with 30.0% who underwent surgical clipping [5].

However, the external validity of randomized trial findings in contemporary real-world populations with aSAH remains a concern. Older patients, those presenting with poor-grade aSAH, and those with concomitant intracerebral hemorrhage (ICH) are often underrepresented in classic trial cohorts [6], although they constitute a substantial proportion of patients treated in routine practice. In this setting, the clinically relevant question extends beyond “EVT versus microsurgery” [7] and instead focuses on identifying the most appropriate securing modality for each patient according to clinical condition and aneurysm morphology. ISAT, for example, predominantly enrolled patients in relatively good clinical condition whose aneurysms were considered suitable for either modality, and its findings cannot necessarily be generalized to all poor-grade or anatomically complex ruptured aneurysms [7,8].

Practically, EVT and microsurgery have complementary advantages. Microsurgery can enable immediate hematoma evacuation and intracranial pressure control when needed and may be advantageous for certain aneurysm locations and configurations [9,10]. Conversely, EVT can avoid surgical exposure and may be preferred for anatomically accessible lesions during clinically vulnerable periods, such as the vasospasm window or in recurrent aneurysms [11,12]. Although current guideline principles support individualized modality selection [3,13], EVT has become the practical first-line option in many centers. Consequently, institutional treatment defaults may influence upfront modality selection, even for aneurysms with features potentially more amenable to microsurgery.

We hypothesized that a shift toward more individualized modality selection would be associated with more favorable functional outcomes. This question is particularly relevant in aging populations and in centers managing a substantial proportion of poor-grade presentations [14,15]. Accordingly, this study aimed to evaluate whether a measurable institutional practice change from an EVT-first default to a modality-neutral individualized pathway was associated with clinical outcomes in routine practice, using a retrospective before-and-after design with fixed time windows.

## 2. Materials and Methods

### 2.1. Study Design and Setting

This single-center, retrospective, before-and-after cohort study was conducted at Iwate Prefectural Central Hospital (Morioka, Japan) to evaluate the clinical outcomes associated with an institutional practice change in upfront modality selection for aSAH. Consecutive patients who underwent aneurysm securing during two pre-specified fixed time windows were included. Two consecutive 15-month periods were analyzed: 1 May 2023 to 31 July 2024 (pre-change) and 1 August 2024 to 31 October 2025 (post-change). The study cohort was derived from a neurosurgical consultation and treatment database rather than from an emergency department–wide registry of all patients presenting with subarachnoid hemorrhage. Except for the institutional practice change in upfront modality selection, the clinical service structure and resource availability remained unchanged during the study period. No institutional changes were made in ICU management, vasospasm surveillance or rescue-treatment protocols, or the availability of EVT and microsurgery. Throughout both periods, our institution had three surgeons capable of performing both EVT and microsurgery; treatment decisions were discussed and determined by these same surgeons, who were also responsible for aneurysm-securing procedures. The post-change categories were used as structured decision considerations to standardize discussion, but they were not rigid rules; final decisions remained individualized and were based on feasibility and overall risk–benefit judgment.

### 2.2. Participants

Consecutive patients with aSAH who underwent aneurysm securing during the study period were included, and no exclusions were applied among eligible patients in this source cohort. Patients who died before neurosurgical consultation, patients without brainstem reflexes who were not considered candidates for aneurysm securing, and patients managed entirely outside the neurosurgical treatment pathway were outside the source database and therefore could not be systematically enumerated in this study.

### 2.3. Treatment Strategy

During the pre-change period, an EVT-first strategy was employed as the default approach for upfront treatment planning. Microsurgery was selected for the following predefined situations: (1) very small aneurysms considered technically unfavorable for EVT; (2) ICH with mass effect (hematoma-related compression that was considered to contribute to deterioration in consciousness or the development of focal neurological deficits); and (3) internal carotid artery (ICA) blister-like aneurysms requiring bypass-assisted trapping.

During the post-change period, the upfront treatment-selection pathway was revised from an EVT-first default to a modality-neutral individualized framework. EVT and microsurgery were considered alternative first-line options, and the securing modality was selected according to clinical condition and aneurysm anatomy, treatment feasibility, and expected invasiveness. Treatment selection in this period was summarized using four decision categories. First, microsurgery was considered clearly indicated when patients met the pre-change microsurgery-exception criteria. Second, microsurgery was favored when aneurysm or hemorrhage characteristics suggested potential advantages of an open approach, including middle cerebral artery aneurysms, ICH not meeting the predefined mass-effect criteria [9,10], broad-neck/low dome-to-neck morphology [16], branch incorporation [17], or otherwise unfavorable endovascular anatomy. Third, EVT was favored for lesions or clinical situations in which an endovascular approach was considered preferable, including treatment during the vasospasm window [11], recurrent aneurysms [18], or posterior circulation aneurysms [3,19]. Fourth, when both modalities were feasible and no clear microsurgery-favoring feature was present, EVT was selected considering its lower procedural invasiveness. These categories were decision considerations rather than rigid treatment rules, and the final treatment was determined according to feasibility and overall risk–benefit judgment.

If EVT was initially intended but was deemed infeasible after anesthesia induction and diagnostic cerebral angiography, the treatment approach was converted to microsurgery. For analysis, patients were classified according to the treatment actually performed. Microsurgery included clipping and, when required, trapping and/or bypass-assisted procedures. EVT referred to coil embolization with adjunctive techniques as needed.

### 2.4. Outcomes

The primary outcome was a favorable functional outcome at 90 days, defined as a modified Rankin Scale (mRS) score of 0–2. The 90-day mRS score was available for all patients, with no missing outcome data. mRS scores were abstracted by a single investigator from routine clinical records. These records had been documented in routine practice by treating physicians. For patients followed at our institution, the 90-day mRS score was obtained from outpatient clinical records. For patients transferred to another hospital, the 90-day mRS score was obtained from clinical records documented by physicians at the receiving institution. The mRS score was directly documented in all cases. Formal blinded outcome adjudication was not performed, and the investigator abstracting outcomes was not formally blinded to treatment period or treatment modality. The full 90-day mRS distribution (0–6) was also analyzed to evaluate the overall shift in disability.

Secondary outcomes and perioperative events included procedure-related complications, delayed cerebral ischemia (DCI) [20], shunt-dependent hydrocephalus, and imaging-confirmed aneurysm recurrence detected within the first 3 months after aneurysm securing. Procedure-related complications were defined as clinically relevant adverse events attributable to aneurysm-securing treatment that occurred intraoperatively or within 30 days after treatment and were considered to contribute to neurological deterioration, require additional treatment or intervention, or affect the 90-day functional outcome. The events actively assessed included intraoperative rupture or re-rupture, thromboembolic events, procedure-related ischemic stroke, parent artery occlusion, access-site complications, postoperative hemorrhage, cerebrospinal fluid leak, wound complications, infection, need for reintervention, and anesthesia-related complications. Minor asymptomatic findings were not counted as procedure-related complications unless they were considered clinically relevant.

In this study, DCI was defined as a new neurological deficit not attributable to other identifiable causes, with radiographic vasospasm evaluated as a related supportive finding when present. Rescue treatments included intrathecal nicardipine and/or endovascular therapy (percutaneous transluminal angioplasty or intra-arterial papaverine hydrochloride administration). Shunt-dependent hydrocephalus was defined as symptomatic hydrocephalus requiring permanent cerebrospinal fluid diversion in patients with ventriculomegaly on imaging and compatible symptoms (e.g., cognitive decline, gait disturbance, and/or urinary incontinence).

Aneurysm recurrence was assessed using follow-up vascular imaging performed within 3 months after aneurysm securing, including magnetic resonance angiography, computed tomography angiography, or digital subtraction angiography. Follow-up vascular imaging was routinely planned within 3 months of aneurysm securing for all treated patients, although occasional deviations may have occurred.

Cerebrospinal fluid drainage (external ventricular or external lumbar drainage) [21,22] and clazosentan use [23] within 0–14 days of symptom onset were also recorded.

### 2.5. Statistical Analysis

Continuous variables are reported as mean (standard deviation [SD]) or median [interquartile range (IQR)], as appropriate, and categorical variables as *n* (%). Between-group comparisons were performed using Welch’s *t*-test for continuous variables and Fisher’s exact test for categorical variables, as appropriate. All tests were two-sided, and *p* < 0.05 was considered statistically significant.

For the primary outcome (90-day mRS 0–2), multivariable logistic regression with Firth penalized likelihood was used as the primary analysis to mitigate small-sample bias and potential separation [24,25]. The exposure of interest was the study period (post-change vs. pre-change). Prespecified covariates were age (per 10-year increase), pre-morbid functional status (pre-mRS ≥ 2), and admission clinical severity (World Federation of Neurosurgical Societies [WFNS] grades IV–V). Adjusted odds ratios (aORs) with 95% confidence intervals (CIs) are reported. As a sensitivity analysis, the model was repeated using conventional (maximum likelihood) logistic regression with the same covariates.

As an exploratory sensitivity analysis, propensity-score matching was performed using the same prespecified covariates as the primary adjusted model. The propensity score was estimated using the study period as the dependent variable and age, pre-morbid mRS ≥ 2, and WFNS grades IV–V as covariates. Patients were matched 1:1 using nearest-neighbor matching without replacement, with a caliper width of 0.2 standard deviations of the logit of the propensity score [26]. Covariate balance after matching was assessed using standardized mean differences [27]. The primary outcome in the matched cohort was compared using an exact McNemar test, and a matched-pair odds ratio was reported.

Additional exploratory sensitivity analyses were performed to evaluate whether the association between the post-change period and favorable outcome was consistent after accounting for hemorrhage severity, aneurysm anatomy, treatment timing, and procedural complexity. Because of the limited number of unfavorable outcome events, a single fully adjusted model including all additional variables was not constructed to reduce overfitting [28,29]. Instead, each additional covariate or covariate domain was added separately to the primary ordinary logistic regression model. These covariates included modified Fisher grades 3–4, ICH on admission, ICH volume, three-category aneurysm location, branch incorporation, broad-neck/low dome-to-neck morphology, admission-to-treatment, procedure duration, and anesthesia duration. Wald 95% CIs and Wald *p* values are reported for the association between the post-change period and favorable 90-day outcome in these models.

For the 90-day mRS shift analysis (0–6), an ordinal logistic regression model under the proportional odds assumption was used, and the adjusted common OR with 95% CI was reported. The proportional odds assumption was assessed using a score test for non-parallel cumulative logits.

Statistical analyses were performed using JMP Student Edition 19 (SAS Institute, Cary, NC, USA). Figures were prepared using Microsoft PowerPoint (Microsoft Corp., Redmond, WA, USA) and GraphPad Prism 10 (GraphPad Software, San Diego, CA, USA).

## 3. Results

### 3.1. Study Population and Treatment Selection

A total of 104 patients with aSAH who underwent aneurysm securing were included in this study (pre-change, *n* = 48; post-change, *n* = 56). All patients in the source cohort were included in the analysis, with no exclusions and no missing data for the primary outcome. Following the institutional practice change in upfront modality selection, the distribution of aneurysm-securing modalities changed substantially. The number of EVT procedures decreased from 38/48 (79.2%) to 21/56 (37.5%), whereas the number of microsurgical procedures increased from 10/48 (20.8%) to 35/56 (62.5%) (*p* < 0.001).

In the post-change period, 16 of the 35 microsurgical cases met the pre-change microsurgery-exception criteria and were classified as microsurgery clearly indicated, whereas the remaining 19 were classified as microsurgery favored under the individualized pathway. Among the 21 EVT-treated patients in the post-change period, EVT was favored in 7 patients, and EVT was selected in 14 patients in whom both modalities were considered feasible, and no clear microsurgery-favoring feature was present.

**Figure 1:**
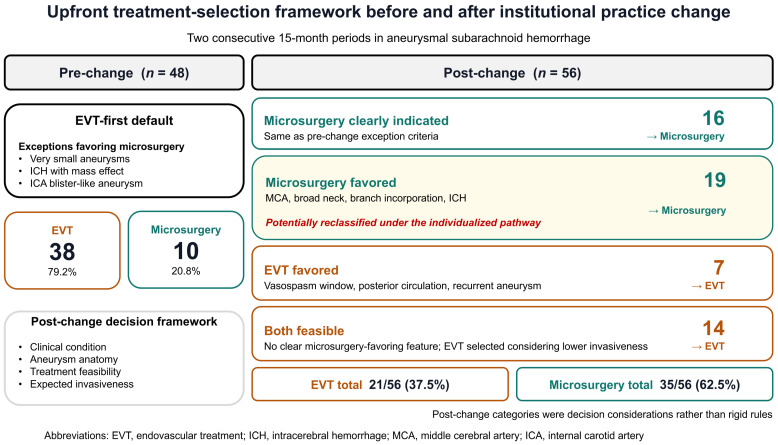
Upfront modality-selection frameworks before and after an institutional practice change in aneurysmal subarachnoid hemorrhage (aSAH). Two consecutive 15-month periods were analyzed: pre-change (1 May 2023 to 31 July 2024; EVT-first) and post-change (1 August 2024 to 31 October 2025; modality-neutral individualized pathway). In the pre-change period, EVT was the default approach, with pre-specified exceptions favoring microsurgery: very small aneurysms with anticipated EVT difficulty, admission ICH with mass effect (hematoma-related compression that was considered to contribute to deterioration in consciousness or the development of focal neurological deficits), and ICA blister-like aneurysms requiring bypass-assisted trapping. In the post-change period, EVT and microsurgery were considered alternative first-line options, and treatment selection was summarized using four decision categories: microsurgery clearly indicated, microsurgery favored, EVT favored, and both modalities feasible. These categories represented decision considerations rather than rigid treatment rules. The figure summarizes the observed final treatment modalities in each period (pre-change: EVT 38/48, microsurgery 10/48; post-change: EVT 21/56, microsurgery 35/56). Among the post-change patients, microsurgery was classified as clearly indicated in 16 patients and favored in 19 patients, whereas EVT was favored in 7 patients and selected in 14 patients in whom both modalities were considered feasible. One post-change patient initially planned for EVT was converted to microsurgery after diagnostic angiography because A2 branch incorporation in an ACom aneurysm was considered to make safe coil embolization infeasible; this patient was analyzed according to the treatment actually performed. Abbreviations: aSAH, aneurysmal subarachnoid hemorrhage; ACom, anterior communicating artery; EVT, endovascular treatment; ICA, internal carotid artery; ICH, intracerebral hemorrhage; MCA, middle cerebral artery.

In one post-change patient, EVT was initially planned but was converted to microsurgery after diagnostic angiography because A2 branch incorporation in an ACom aneurysm was considered to make safe coil embolization infeasible. This patient was analyzed according to the treatment actually performed. Descriptively, 19 of 56 post-change patients (33.9%), corresponding to 19 of 35 microsurgically treated patients (54.3%), did not meet the pre-change microsurgery-exception criteria but were treated with microsurgery because microsurgery-favoring features were identified under the individualized pathway (Figure 1).

### 3.2. Baseline Characteristics

The baseline patient characteristics are presented in Table 1. Detailed clinical, anatomical, timing, and procedural variables are provided in Supplementary Table S1. The key baseline variables retained in the main table were broadly comparable between the two periods. Age [65.5 (15.0) vs. 65.4 (14.1) years; *p* = 0.960] and female sex [36/48 (75.0%) vs. 42/56 (75.0%); *p* = 1.000] were similar between the pre-change and post-change cohorts. Admission severity measures also did not differ significantly, including WFNS grades IV–V [14/48 (29.2%) vs. 23/56 (41.1%); *p* = 0.225], modified Fisher grades 3–4 [30/48 (62.5%) vs. 30/56 (53.6%); *p* = 0.427], and ICH on admission [15/48 (31.2%) vs. 13/56 (23.2%); *p* = 0.383]. Aneurysm-related treatment-selection variables retained in the main table were also similar between periods, including broad aneurysm location classified as anterior versus posterior circulation (*p* = 1.000), broad-neck/low dome-to-neck morphology [32/48 (66.7%) vs. 40/56 (71.4%); *p* = 0.672], and branch incorporation [5/48 (10.4%) vs. 4/56 (7.1%); *p* = 0.729].

In Supplementary Table S1, vascular risk factors, exact aneurysm sites, aneurysm dome size, neck width, dome-to-neck ratio, ICH volume among patients with ICH, mass effect, acute hydrocephalus, and treatment during the vasospasm window were also generally comparable between periods. Aneurysm securing within 24 h from admission was achieved in nearly all patients in both periods [47/48 (97.9%) vs. 55/56 (98.2%); *p* = 1.000]. Admission-to-treatment [3.9 (2.9–5.9) vs. 6.6 (3.9–14.9) hours; *p* < 0.001], procedure duration [174 (124–256) vs. 232 (190–315) minutes; *p* = 0.003], and anesthesia duration [236 (185–330) vs. 316 (248–407) minutes; *p* = 0.001] were longer in the post-change period. Before the 90-day assessment, 22/48 patients (45.8%) in the pre-change period and 35/56 patients (62.5%) in the post-change period had been transferred to another institution (*p* = 0.114). The number of receiving institutions was 6 in the pre-change period and 13 in the post-change period.

### 3.3. Primary Outcome (90-Day mRS 0–2)

A favorable functional outcome at 90 days (mRS 0–2) was observed in 25/48 (52.1%) patients in the pre-change period and 36/56 (64.3%) patients in the post-change period (*p* = 0.235). In the prespecified multivariable analysis using Firth penalized logistic regression (adjusted for age per 10-year increase, pre-morbid mRS ≥ 2, and admission WFNS grades IV–V), the post-change period was associated with higher odds of a favorable outcome (aOR 3.82, 95% CI 1.31–12.79; *p* = 0.009) (Table 2). Findings were directionally consistent in the sensitivity analysis using conventional logistic regression (aOR 4.41, 95% CI 1.43–15.95; *p* = 0.009). Older age (per 10-year increase) and admission WFNS grades IV–V were also associated with lower odds of a favorable outcome (Table 2).

Propensity-score matching using the same prespecified covariates as the primary adjusted model yielded 45 matched pairs with excellent covariate balance. In the matched cohort, favorable 90-day outcomes occurred in 32/45 patients (71.1%) in the post-change period and 23/45 patients (51.1%) in the pre-change period (matched-pair OR 4.00, 95% CI 1.08–22.09; *p* = 0.035), supporting the direction of the primary adjusted analysis. Details of the propensity-score matched analysis are provided in Supplementary Table S2.

Additional exploratory sensitivity analyses that separately accounted for hemorrhage severity, aneurysm anatomy, treatment timing, and procedural complexity were also directionally consistent with the primary analysis (Supplementary Table S3).

### 3.4. Shift Analysis of 90-Day mRS (0–6)

**Figure 2:**
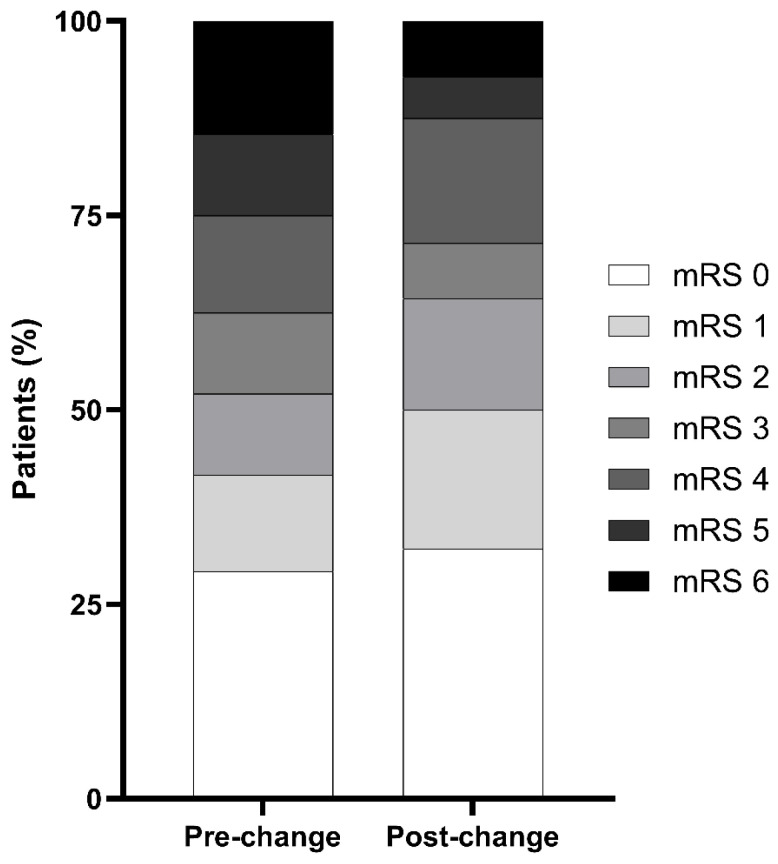
Distribution of 90-day modified Rankin Scale scores before and after the institutional practice change. Stacked bar charts show the distribution of 90-day mRS scores (0–6) in the pre-change (*n* = 48) and post-change (*n* = 56) cohorts. The between-period shift was assessed using an adjusted proportional odds model (ordinal logistic regression) with covariates for age (per 10-year increase), pre-morbid mRS ≥ 2, and admission WFNS grades IV–V. Abbreviations: mRS, modified Rankin Scale; WFNS, World Federation of Neurosurgical Societies.

**Figure 3:**
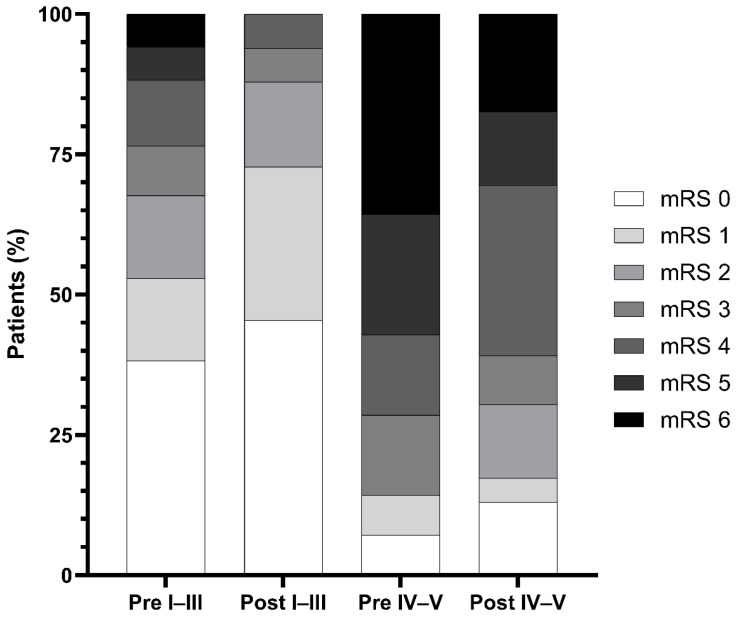
Distribution of 90-day mRS scores by admission WFNS grade and study period. Stacked bar charts show the distribution of 90-day modified Rankin Scale (mRS) scores (0–6) in the pre-change (2023–2024) and post-change (2024–2025) cohorts stratified by World Federation of Neurosurgical Societies (WFNS) grade (I–III vs. IV–V). Bars represent the proportion of patients within each stratum (pre-change: WFNS I–III, *n* = 34; WFNS IV–V, *n* = 14; post-change: WFNS I–III, *n* = 33; WFNS IV–V, *n* = 23). Abbreviations: mRS, modified Rankin Scale; WFNS, World Federation of Neurosurgical Societies.

The distribution of 90-day mRS scores (0–6) is shown in Figure 2. In an adjusted proportional odds model (ordinal logistic regression) including age (per 10-year increase), admission WFNS grades IV–V, and pre-morbid mRS ≥ 2, the post-change period was associated with an overall shift toward better functional outcome compared with the pre-change period (adjusted common OR 2.36, 95% CI 1.15–4.91; *p* = 0.021). The proportional odds assumption was not violated (score test for non-parallel cumulative logits: χ^2^ = 15.84, df = 20, *p* = 0.727). A descriptive subgroup analysis stratified by admission WFNS grade is shown in Figure 3. Among patients with WFNS grades I–III, favorable outcomes (mRS 0–2) were numerically more frequent in the post-change period (29/33 [87.9%] vs. 23/34 [67.6%]; *p* = 0.077). Among patients with WFNS grades IV–V, favorable outcomes were also numerically more frequent (7/23 [30.4%] vs. 2/14 [14.3%]; *p* = 0.434).

### 3.5. Secondary Outcomes and Procedure-Related Complications

The secondary outcomes and perioperative events are summarized in Table 3. No statistically significant between-period differences were observed in any secondary outcome or perioperative event. Procedure-related complications occurred in 7/48 patients (14.6%) in the pre-change period and 6/56 patients (10.7%) in the post-change period (*p* = 0.569). Similarly, no significant differences were observed in DCI (8/48 [16.7%] vs. 7/56 [12.5%]; *p* = 0.586), cerebrospinal fluid drainage within 0–14 days after symptom onset (30/48 [62.5%] vs. 39/56 [69.6%]; *p* = 0.533), clazosentan use within 0–14 days after symptom onset (34/48 [70.8%] vs. 41/56 [73.2%]; *p* = 0.829), shunt-dependent hydrocephalus (13/48 [27.1%] vs. 16/56 [28.6%]; *p* = 1.000), or imaging-confirmed aneurysm recurrence detected within the first 3 months of follow-up (7/48 [14.6%] vs. 4/56 [7.1%]; *p* = 0.338). The distribution of procedure-related complication types is provided in Supplementary Table S4.

## 4. Discussion

In this single-center retrospective before-and-after study, an institutional practice change from an EVT-first approach to a modality-neutral individualized upfront treatment-selection pathway was associated with more favorable adjusted 90-day functional outcomes in patients with aSAH, alongside an increased proportion of microsurgery. The findings were further supported directionally by propensity-score matching and by multiple exploratory sensitivity analyses. However, the unadjusted comparison did not reach statistical significance, and these results should therefore be interpreted as hypothesis-generating rather than as evidence of a causal effect.

Since the publication of the International Subarachnoid Aneurysm Trial [1,2], EVT has become increasingly common in the treatment of aSAH. Current guideline principles support early aneurysm securing and individualized modality selection based on expertise in both endovascular and microsurgical treatment [3,13]; nevertheless, EVT is used as the first-line treatment for aSAH in many real-world settings [5]. The present study does not establish individualized treatment selection as a new concept. Rather, it pragmatically evaluates the local implementation of a change from an EVT-first institutional default to a modality-neutral individualized strategy in a contemporary real-world cohort.

When interpreting these results, the difference between the unadjusted and adjusted analyses should be considered carefully. Although baseline characteristics were broadly similar between periods, the post-change cohort included a higher proportion of patients with poor-grade presentations, which may have biased the unadjusted comparison toward worse outcomes. Adjustment for prespecified prognostic factors, including age, admission WFNS grade, and pre-morbid function, may have reduced part of this imbalance and clarified the association between study period and outcome. Propensity-score matched analysis using the same prespecified covariates was directionally consistent with the primary Firth logistic regression model, and additional sensitivity analyses that separately accounted for modified Fisher grade, ICH, aneurysm location, aneurysm morphology, timing, and procedural duration yielded consistent estimates. Nevertheless, these analyses cannot eliminate residual or unmeasured confounding, and the adjusted estimates should be interpreted cautiously given the modest sample size and limited number of outcome events.

Treatment timing and procedural variables are also important potential confounders in before-and-after studies of aSAH [3,13]. In the present cohort, aneurysm securing within 24 h from admission was achieved in nearly all patients in both periods, whereas admission-to-treatment, procedure duration, and anesthesia duration were longer in the post-change period. These longer timing and procedural parameters likely reflected, at least in part, the increased proportion of microsurgical aneurysm-securing procedures in the post-change period, as microsurgery generally requires additional operative preparation and longer procedural and anesthetic times than EVT. Thus, the observed association is unlikely to be explained simply by shorter treatment delays or shorter procedures in the post-change period.

No statistically significant between-period differences were observed in individual secondary outcomes, including procedure-related complications, DCI, CSF drainage, clazosentan use, shunt-dependent hydrocephalus, and aneurysm recurrence. This finding suggests that the association between the post-change period and more favorable adjusted functional outcomes may not be explained by a single measurable downstream complication. However, given the observational design and limited sample size, this interpretation should remain cautious. More individualized modality selection may have improved the alignment between patient condition, aneurysm anatomy, hemorrhage-related factors, and the selected aneurysm-securing modality, but this mechanism cannot be directly established from the present data.

The subgroup findings shown in Figure 3 should also be interpreted cautiously. Among patients with WFNS grades I–III and those with WFNS grades IV–V, favorable outcome was numerically more frequent in the post-change period. However, these analyses were descriptive and based on relatively small numbers and therefore, should not be overinterpreted.

This study has several limitations. First, as a retrospective single-center before-and-after study, the results may have been influenced by secular trends, time-varying co-interventions, and unmeasured confounding factors, and generalizability may vary across institutions. Although consecutive patients were included within prespecified fixed time windows, and no formal institutional changes were made in ICU management, vasospasm surveillance or rescue-treatment protocols, or the availability of EVT and microsurgery, time-dependent changes in ICU care, perioperative management, endovascular practice, or team experience cannot be fully excluded. Second, although 90-day mRS scores were available for all patients and were directly documented, functional outcomes were abstracted from routine clinical records rather than assessed through formal, blinded adjudication. In addition, a substantial proportion of patients were transferred to other institutions before the 90-day assessment, which may have introduced heterogeneity in outcome ascertainment. Third, the sample size was limited, and the precision and stability of the adjusted estimates are therefore constrained. In the additional sensitivity analyses, each additional covariate or covariate domain was added separately to avoid overfitting, and simultaneous adjustment for all clinical and anatomical factors was not feasible. Fourth, procedure duration and anesthesia duration may lie on the treatment pathway and should therefore be interpreted exploratorily.

## 5. Conclusions

In this contemporary real-world aSAH cohort, a measurable institutional practice change from an EVT-first default to a modality-neutral individualized upfront treatment-selection pathway was associated with more favorable adjusted 90-day functional outcomes. These findings support the clinical relevance of implementing guideline-consistent individualized modality selection in routine aSAH care, but should be interpreted as hypothesis-generating given the retrospective single-center before-and-after design. Confirmation in multicenter studies is warranted.

## Figures and Tables

**Table 1:** Baseline characteristics.

Characteristics	Pre-Change (*n* = 48)	Post-Change (*n* = 56)	*p* Value
Age, mean (SD), years	65.5 (15.0)	65.4 (14.1)	0.960
Female sex	36 (75.0%)	42 (75.0%)	1.000
WFNS grades IV–V	14 (29.2%)	23 (41.1%)	0.225
Modified Fisher grades 3–4	30 (62.5%)	30 (53.6%)	0.427
ICH on admission	15 (31.2%)	13 (23.2%)	0.383
Aneurysm location			1.000
Anterior circulation	45 (93.8%)	53 (94.6%)	
Posterior circulation	3 (6.2%)	3 (5.4%)	
Broad-neck/low dome-to-neck morphology	32 (66.7%)	40 (71.4%)	0.672
Branch incorporation	5 (10.4%)	4 (7.1%)	0.729
Treatment modality			<0.001
Endovascular treatment	38 (79.2%)	21 (37.5%)	
Microsurgery	10 (20.8%)	35 (62.5%)	

Notes: Values are presented as *n* (%) unless otherwise specified. The pre-change and post-change periods were 1 May 2023–31 July 2024 and 1 August 2024–31 October 2025, respectively. *p* values were calculated using Welch’s *t*-test for age and Fisher’s exact test for categorical variables. Broad-neck/low dome-to-neck morphology was defined as neck width ≥ 4 mm or dome-to-neck ratio < 2. EVT, endovascular treatment; ICH, intracerebral hemorrhage; WFNS, World Federation of Neurosurgical Societies.

**Table 2:** Multivariable logistic regression for 90-day favorable outcomes.

Variable	Primary Analysis (Firth) aOR (95% CI)	*p* Value	Sensitivity Analysis (Conventional) aOR (95% CI)	*p* Value
Study period	3.82 (1.31–12.79)	0.009	4.41 (1.43–15.95)	0.009
Age	0.43 (0.25–0.66)	<0.001	0.39 (0.22–0.63)	<0.001
Pre-morbid mRS	1.12 (0.14–7.79)	1.000	1.06 (0.11–8.31)	0.955
WFNS grade	0.05 (0.01–0.15)	<0.001	0.03 (0.01–0.12)	<0.001

Notes: The outcome was a 90-day favorable outcome, defined as a modified Rankin Scale score of 0–2. The pre-change and post-change periods were 1 May 2023–31 July 2024 and 1 August 2024–31 October 2025, respectively. All estimates are mutually adjusted for the variables listed in the table. Contrasts were as follows: study period, post-change vs. pre-change; age, per 10-year increase; pre-morbid mRS, ≥2 vs. 0–1; and WFNS grade, IV–V vs. I–III. The primary analysis used Firth penalized logistic regression. The sensitivity analysis used ordinary maximum-likelihood logistic regression with the same covariates. The total sample size was 104, and the number of favorable outcomes was 61 (pre-change, 25/48; post-change, 36/56). aOR, adjusted odds ratio; CI, confidence interval; mRS, modified Rankin Scale; WFNS, World Federation of Neurosurgical Societies.

**Table 3:** Secondary outcomes and procedure-related complications.

Outcome	Pre-Change (*n* = 48)	Post-Change (*n* = 56)	*p* Value
Procedure-related complications (any)	7 (14.6%)	6 (10.7%)	0.569
Delayed cerebral ischemia	8 (16.7%)	7 (12.5%)	0.586
CSF drainage (EVD or ELD)	30 (62.5%)	39 (69.6%)	0.533
Clazosentan use	34 (70.8%)	41 (73.2%)	0.829
Shunt-dependent hydrocephalus	13 (27.1%)	16 (28.6%)	1.000
Aneurysm recurrence	7 (14.6%)	4 (7.1%)	0.338

Notes: Values are presented as *n* (%). The pre-change and post-change periods were 1 May 2023–31 July 2024 and 1 August 2024–31 October 2025, respectively. *p* values were calculated using two-sided Fisher’s exact test. Procedure-related complications were defined as clinically relevant adverse events attributable to aneurysm-securing treatment that occurred intraoperatively or within 30 days after treatment. Delayed cerebral ischemia was assessed during hospitalization. CSF drainage and clazosentan use were recorded within 0–14 days from symptom onset. Shunt-dependent hydrocephalus was defined as symptomatic hydrocephalus requiring permanent CSF diversion. Aneurysm recurrence was defined as imaging-confirmed recurrence detected within 3 months after aneurysm securing. CSF, cerebrospinal fluid; ELD, external lumbar drainage; EVD, external ventricular drainage.

## Data Availability

The data presented in this study are available from the corresponding author on reasonable request. The data are not publicly available because of privacy and institutional restrictions.

## Supplementary Materials

The following supporting information can be downloaded at: https://www.mdpi.com/article/10.3390/neurolint18050093/s1, Supplementary Table S1: Detailed baseline, aneurysm, and timing / procedural characteristics not shown in the main table; Supplementary Table S2: Propensity-score matched sensitivity analysis; Supplementary Table S3: Exploratory sensitivity analyses for the primary outcome; Supplementary Table S4: Distribution of procedure-related complication types.
