# Supplementary material for: Individualized Upfront Treatment Selection for Aneurysmal Subarachnoid Hemorrhage and Functional Outcomes: A Single-Center Retrospective Before-and-After Cohort Study
Source: Neurol Int. 2026 May 15;18(5):93. doi: 10.3390/neurolint18050093 (PMC13210212; doi:10.3390/neurolint18050093)
Supplement: Supplementary file 1 [file neurolint-18-00093-s001.zip › neurolint-4281386-supplementary.pdf]

Supplementary Materials

Supplementary Table S1. Detailed baseline, aneurysm, and timing / procedural characteristics not shown in the main table.

Characteristics	Pre-change (n = 48)	Post-change (n = 56)	p value
Vascular risk factors			
Current smoker	16 (33.3%)	20 (35.7%)	0.839
Hypertension	20 (41.7%)	22 (39.3%)	0.843
Dyslipidemia	12 (25.0%)	9 (16.1%)	0.329
Diabetes mellitus	2 (4.2%)	7 (12.5%)	0.172
Aneurysm morphology and location			
Aneurysm dome size, mm	5.3 [4.1–6.4]	4.8 [3.5–7.2]	0.669
Aneurysm neck width, mm	2.8 [2.3–3.9]	2.8 [2.3–3.5]	0.906
Dome-to-neck ratio	1.7 [1.5–2.2]	1.7 [1.4–2.1]	0.696
Aneurysm location			0.712
ACom	12 (25.0%)	20 (35.7%)	
Distal ACA	3 (6.2%)	4 (7.1%)	
MCA	15 (31.2%)	13 (23.2%)	
IC-PCom	13 (27.1%)	11 (19.6%)	
IC-AChA	0 (0.0%)	2 (3.6%)	
IC anterior wall	2 (4.2%)	2 (3.6%)	
IC top	0 (0.0%)	1 (1.8%)	
PICA	1 (2.1%)	0 (0.0%)	
AICA	1 (2.1%)	2 (3.6%)	
BA tip	1 (2.1%)	0 (0.0%)	
BA-SCA	0 (0.0%)	1 (1.8%)	
Admission imaging and timing / procedural variables			
ICH volume, mL among ICH patients	9.1 [1.4–27.2]	28.4 [7.7–62.4]	0.180
Mass effect on admission	4 (8.3%)	9 (16.1%)	0.373
Acute hydrocephalus on admission	4 (8.3%)	8 (14.3%)	0.377
Treated during vasospasm window, days 4–14	4 (8.3%)	2 (3.6%)	0.411
Aneurysm securing within 24 h from admission	47 (97.9%)	55 (98.2%)	1.000

Characteristics	Pre-change (n = 48)	Post-change (n = 56)	p value
Admission-to-treatment, hours	3.9 [2.9–5.9]	6.6 [3.9–14.9]	<0.001
Procedure duration, min	174 [124–256]	232 [190–315]	0.003
Anesthesia duration, min	236 [185–330]	316 [248–407]	0.001
Transfer status before 90-day assessment			
Number of receiving institutions	6	13	
Transferred to another institution before 90 days	22 (45.8%)	35 (62.5%)	0.114

Notes: Values are presented as n (%) or median [IQR] unless otherwise specified. Variables retained in the main Table 1 are not repeated here. The pre-change and post-change periods were 1 May 2023–31 July 2024 and 1 August 2024–31 October 2025, respectively. Mass effect was defined as hematoma-related compression that was considered to contribute to deterioration in consciousness or the development of focal neurological deficits. Acute hydrocephalus on admission was defined as hemorrhage-related obstructive hydrocephalus requiring external ventricular drainage. Admission-to-treatment was defined as the interval from hospital admission to the start of aneurysm securing. Procedure and anesthesia durations are shown for all procedures combined, without stratification by treatment modality. P values were calculated using Welch's t-test for continuous variables and Fisher's exact test for categorical variables, as appropriate. For patients followed at our institution, the 90-day mRS score was obtained from outpatient clinical records. For patients transferred to another hospital, the 90-day mRS score was obtained from clinical records documented by physicians at the receiving institution. The mRS score was directly documented in all cases. Formal blinded outcome adjudication was not performed, and the investigator abstracting outcomes was not formally blinded to treatment period or treatment modality. The number of receiving institutions indicates unique centers within each period and was not subjected to statistical testing. ACA, anterior cerebral artery; AChA, anterior choroidal artery; ACom, anterior communicating artery; AICA, anterior inferior cerebellar artery; BA, basilar artery; IC, internal carotid; ICH, intracerebral hemorrhage; MCA, middle cerebral artery; PCom, posterior communicating artery; PICA, posterior inferior cerebellar artery; SCA, superior cerebellar artery.

Supplementary Table S2. Propensity-score matched sensitivity analysis

Item	Pre-change	Post-change
Cohort		
Original cohort	48	56
Matched cohort	45	45

Unmatched patients	3	11
Covariate balance after matching (SMD)		
Age	0.006	
Pre-morbid mRS ≥2	0.000	
WFNS grades IV–V	0.000	
Favorable 90-day outcome (mRS 0–2)	23/45 (51.1%)	32/45 (71.1%)
Discordant pairs	3	12
Matched-pair OR (95% exact CI)	4.00 (1.08–22.09)	
P value	0.035	

Notes: Values are presented as n (%) unless otherwise specified. Propensity-score matching was performed using age, pre-morbid mRS ≥2, and WFNS grades IV–V. Patients were matched 1:1 using nearest-neighbor matching without replacement, with a caliper width of 0.2 standard deviations of the logit of the propensity score. The matched-pair OR was calculated from discordant pairs favoring the post-change period versus the pre-change period (12 pairs in which only the post-change patient had a favorable outcome and 3 pairs in which only the pre-change patient had a favorable outcome); the 95% exact CI was obtained by exact binomial inversion, and the P value was calculated using the exact McNemar test. SMD indicates the standardized mean difference after matching. CI, confidence interval; mRS, modified Rankin Scale; OR, odds ratio; SMD, standardized mean difference; WFNS, World Federation of Neurosurgical Societies.

Supplementary Table S3. Exploratory sensitivity analyses for the primary outcome

Model	Additional covariate/domain	EPV	aOR (95% CI) for post-change period	*p* value
Primary + modified Fisher grade	Modified Fisher grades 3–4	7.2	4.54 (1.24–16.70)	0.023
Primary + ICH on admission	ICH on admission	7.2	3.85 (1.14–12.98)	0.030
Primary + ICH volume	ICH volume per 10 mL	7.2	4.69 (1.39–15.82)	0.013
Primary + aneurysm location	Three-category aneurysm location	6.1	4.53 (1.31–15.66)	0.017
Primary + branch incorporation	Branch incorporation	7.2	4.44 (1.34–14.69)	0.015
Primary + broad-neck morphology	Broad-neck / low dome-to-neck morphology	7.2	4.41 (1.34–14.50)	0.015

Primary + admission-to-treatment	admission-to-treatment per 1 h	7.2	5.34 (1.45–19.57)	0.012
Exploratory + procedure duration	Procedure duration per 60 min	7.2	5.22 (1.51–18.09)	0.009
Exploratory + anesthesia duration	Anesthesia duration per 60 min	7.2	5.15 (1.49–17.85)	0.010

Notes: The outcome was a 90-day favorable outcome, defined as a modified Rankin Scale score of 0–2. The primary ordinary logistic model included study period, age per 10-year increase, pre-morbid mRS ≥2, and admission WFNS grades IV–V. Each sensitivity model added the listed covariate or covariate domain one at a time. Ordinary maximum-likelihood logistic regression was used, and the 95% confidence intervals and P values shown in the table are Wald estimates for the association between the post-change period and favorable 90-day outcome in each model. EPV was calculated as 43 unfavorable outcomes divided by the number of model parameters, including the intercept; a single fully adjusted model including all additional variables was avoided to reduce overfitting. ICH volume was coded as 0 mL for patients without ICH. Three-category aneurysm location comprised MCA, posterior circulation, and other anterior circulation aneurysms; other anterior circulation aneurysms included ACA/ACom and ICA aneurysms and served as the reference category. Broad-neck / low dome-to-neck morphology was defined as neck width ≥4 mm or dome-to-neck ratio <2. Admission-to-treatment was analyzed per 1-h increase. Models including procedure or anesthesia duration were considered exploratory because these variables may lie on the treatment pathway. aOR, adjusted odds ratio; ACA, anterior cerebral artery; ACom, anterior communicating artery; CI, confidence interval; EPV, events per parameter; ICA, internal carotid artery; ICH, intracerebral hemorrhage; MCA, middle cerebral artery; mRS, modified Rankin Scale; WFNS, World Federation of Neurosurgical Societies.

Supplementary Table S4. Distribution of procedure-related complication types.

Outcome	Pre-change (*n* = 48)	Post-change (*n* = 56)
Re-rupture	3 (6.3%)	1 (1.8%)
Procedure-related ischemic stroke with neurological deficit (non-DCI)	2 (4.2%)	3 (5.4%)
Myocardial infarction	1 (2.1%)	0 (0%)
Cerebrospinal fluid leak	0 (0%)	1 (1.8%)
Infection (meningitis suspected)	1 (2.1%)	1 (1.8%)
Any procedure-related complication	7 (14.6%)	6 (10.7%)

Notes: Values are presented as n (%). Complication subtypes were summarized descriptively. Re-rupture was assessed intraoperatively or during hospitalization. Procedure-related ischemic stroke with neurological deficit, myocardial infarction, cerebrospinal fluid leak, infection, and any procedure-related complication were assessed intraoperatively or within 30 days after aneurysm securing, as applicable. Infection was defined as fever (≥38 °C) with CSF findings consistent with meningitis and antibiotic treatment.

Procedure-related ischemic stroke (non-DCI) indicates a new infarction with a neurological deficit attributed to the securing procedure and not meeting the study definition of DCI.
